# The Hospitals

**Published:** 1874-08

**Authors:** 


### The Hospitals.

***Cook County Hospital.*** Surgical Clinic. May 26. Service of Dr. Bogue. Reported for Chicago Medical Journal by Ralph E. Starkweather, M.D.

*Osteo-Aneurism of the Right Ilium.*

W. B., a laborer, 22 years of age, while running backwards, in August last, fell heavily, striking on his back and right buttock. In December, a lump appeared in the gluteal region, and became painful—this gradually increased in size; the pain extended down the right thigh. The tumor is ovoid, the size of a very large egg, its centre is over the sacro iliac symphysis, the size increased two inches in the past four days. It pulsates, but no bruit can be perceived. Upon explorative puncture, the patient lying prone, the needle was inserted three and one-half inches upwards and forwards at the border of the tumor; blood came per saltum—synchronous with the pulse, but did not so continue at all depths.

At another time, upon a different site and angle of puncture, a cavity was discovered. At first, the blood was dark venous; deeper down, came the cavity from which blood flowed per saltum; deeper still, a bony base was felt, and there was no flowing of the blood. Pressure on the abdominal aorta lessened the tension of the tumor, and stopped its pulsations, but did not alter the uneven feeling of the tumor. A fluid drachm of liq. ferri persulphatis was injected, causing the pulsations to cease; and morphine was ordered as required.

Subsequently, on May 30, Dr. Bogue, by an incision of over four inches, laid open the tumor and turned out its contents—a mass of small anastomosing vessels and erectile tissue, resting upon the eroded inner plate of the ilium. The bone had degenerated; a roughened collar of bone could be felt, circumscribing the base of the tumor, encroaching on the edge of the sacrum. The glavano-cautery was applied to two large vessels and the lips of the wound; the cavity, which would admit the whole hand, was packed with lint and the dried sulphate of iron.

Dr. Bogue remarked, that it was rare that a contused wound is followed by an aneurism. Sometimes an incised wound does produce accidentally an aneurism. It is probable that this aneurismal tumor is of malignant character.

CLINIC, by Dr. LYMAN. June 16.

A case of chronic sub-acute meningitis, due to a fall received three months ago, by which the head was injured. Complains of pain over occiput and the temples; pupils dilated; pulse sixty, and weak. The right hand raises the dynamometer 102°; the left to 110°. Ordered counter-irritation; opium as anodyne; potassium bromide as alterative.

A case of pneumo hydro thorax, in which the dullness extended on the right side, posteriorly, as high as the third intercostal space. By means of the aspirator, ninety ounces of a clear, lemon-colored fluid was drawn off, the puncture being made at the seventh intercostal space. The patient should sit up during this operation, and be warned that there may, for various reasons, be danger of collapse of the lung and chest wall. It is well to strap the side of the chest.

A case of sciatica, in a man forty years of age, due to overwork, mal-nutrition and exhaustion. Generally this affection is of rheumatic origin, or may be due to exostosis, to syphilis, or to cancerous growths. Ordered first a laxative, then cod-liver oil, tinct. ferri muriatis, and a grain of quinine three times a day; also anodynes. Dr. Lyman remarked that the profession was becoming more cautious in the use of morphine by hypodermic injection. Chloroform, once in four or five thousand administrations, would cause a death. Hence in this case he used the endermic method, and showed the class that it would act promptly, and produce none of the discomforts which opium may occasion. Taking a thimble he filled it with lint and soaked it with strong liquor ammonia. Removing the cuticle, he applied the one-third of a grain of morphia sulphate, covered it with a pledget of lint, then a piece of oiled silk, fixing it with strips of adhesive plaster. Cantharidis should not be used as a vesicant for this purpose.

SURGICAL CLINIC, by Dr. BOGUE. June 16.

A boy, ten years of age, while trying to jump on to a train of cars in motion, fell, and sustained a compound and badly comminuted fracture of the foot and lower third of right leg. Amputation under ether was done at the point of election below the knee. Anterior and posterior flaps were made. The hæmorrhage was slight, Esmarch's method being used.

An excision at the right ankle joint was made in the case of a laborer who had fallen down into a cellar, producing a fracture of the fibula, its lower two inches; also of the lower and outer end of the tibia obliquely downward, forward and inward; also a comminuted fracture of the astragalus, together with laceration of the ligaments of the joint. The rubber tubing was adjusted to the limb, and retained for over seventy minutes. The patient being fully under the influence of ether, the first incision was made along the posterior border of the fibula, behind the external malleolus to the fifth metatarsal bone. A similar incision was made on the inner aspect of the limb; portions of the fibula and tibia were removed, and the whole of the astragalus; the articular cartilage of the os calcis was removed. After ligaturing four vessels, an obstinate capillary oozing was checked by the dried powdered persulphate of iron. The limb, joint and foot were encased in a plaster of Paris dressing.

Medical Clinic, by Dr. H. M. Lyman. June 19.

A young colored woman was under treatment for phthisis pulmonalis, complicated with Bright's disease of the kidney.

*Unilateral Rheumatism, followed by Chorea of the opposite side.*

The patient, a girl of fifteen, had suffered from rheumatism, the joints and limbs of the left side being the seat of pain and swelling, for more than one month. At present she lies in bed unable to walk, with twitchings of the right leg—no power in the hand or ankle of same side. Mental condition very much weakened; the choraic movements very strong and marked on the right half of the body.

An English writer, Dr. Hughlings Jackson, speaking of chorea as a disease, the sequence of rheumatism, advances the theory that the valves of the heart become covered by vegetations, the result of inflammatory rheumatism; some of these are detached, and being carried by the current of blood, lodge in the corpus striatum of the brain, and hence chorea results; the shortest distance between the heart and brain would be upon the left side, and hence the lesion would be shown upon the right side of the body, as is the case in this choraic girl. The prognosis is usually not fatal, unless there is loss of sleep. Treatment may be, quinine, strychnia (gr. $\frac{1}{60}$), arsenic, cod-liver oil. Chloral will check the restlessness.

SURGICAL CLINIC, by Dr. BOGUE. June 23.

A case of synovitis of the right knee in a little girl six years of age, is treated, in its present stage, by straps of adhesive plaster and bandaging.

Congenital malformation of the right external ear, in a boy eight months of age. The auricle was no more than one-third the size of that of the opposite ear, and the concavity was directed backwards, the convexity of the entire rim of the helix pointed forward towards the face; the middle and internal ears were thought to be normal. No treatment was, for the present, advised.

Dr. Bogue laid open several fistulous tracts in a stump formed after an amputation of the left thigh at the hip joint. The operation on this man was done one year ago, and was the second that had been done in this hospital, each with successful results.

SURGICAL CLINIC, by Dr. BOGUE. June 30. *Syphilitic Pemphigus and Lupus—Two Cases Simple Fracture of the Clavicle—Dislocation of the Right Shoulder—Fracture of the Leg.*

The patient with syphilis said he had contracted a chancre fifteen months ago, and been very improperly treated, to which fact he attributes his present truly unfortunate and deplorable condition. His entire forehead is a mass of pustulation and crusts of pemphigus. The crusts are a red-brown color, roughened and furrowed, and much larger in size than those of rupia. There are similar patches upon each malar prominence. A line of these crusts extends from the forehead down upon, and on either side of, the ridge of the nose. The larger part of the outer wall of the nose has been destroyed, and the floor of the nose is left exposed. All portions of his body are covered with scars from various syphilitic eruptions. The patient has a bad cough, and is in a very depressed, anemic, emaciated condition. Dr. Bogue ordered tr. cinchona comp. and the syrup iodide of iron, and said that in this case, for the present, the treatment would be such as would improve the general condition, and, later, a treatment for syphilis, the mild use of hydrargyrum, not long continued, and in small quantity. Then some form of iodine.

A blacksmith, twenty-four years of age, had his left clavicle broken at its outer third by a blow. This fracture was treated by keeping the man in bed, and so placing a pillow under the right shoulder that the weight and position of the joint and extremity on the injured side would keep the points of fracture in line and apposition. Several other methods were spoken of, such as using a sling, or adhesive plaster, or Fox's apparatus. In the case of a lady patient, where more attention to a possible deformity must be paid, Dr. Bogue would use Taylor's splint for posterior deformity of the spine.

*Dislocation of the Right Shoulder.*

The patient had fallen out of a wagon, the week previous, and had been treated for simple contusion and sprain. The dislocation was downwards, and was reduced, under ether, by pulling the arm outwards and upwards. Afterwards the forearm was brought across the chest, and retained by a bandage.

*Fracture of the Left Leg.*

A young man of twenty-two years of age, had broken his leg in two places, the tibia four inches from the internal malleolus, and the fibula two inches below its head. There was no displacement. The plaster of Paris dressing was applied thirty-six hours after the injury was received.

---

***The Alexian Hospital.*** Service of Dr. A. J. BAXTER. July 1.

At present, there are under the care of the Alexian Brotherhood, forty patients, though the hospital has a capacity for one hundred and fifty patients. There is a noticeable absence in the wards of the crowd of hospital tramps and "revolvers," candidates for the almshouse and lunatic asylums, old pensioners and chronic ulcer impostors.

*Lithotomy—Compound Fracture—Strumous Disease of the Knee, and Amputation—Conical Stump—Esmarch's Bloodless Method.*

Dr. Baxter removed a vesical calculus, weighing one and one-half ounces, from a boy of thirteen, by lateral lithotomy; the calculus was attached to a very unusual place, being almost encysted behind the symphysis pubis.

A case of compound and comminuted fracture of the leg was progressing finely. A blanket, being so folded as to form a case and splint for the limb, strengthened by thin wooden splints placed in the folds on either side, and cold water dressings, were the only treatment. A plaster of Paris splint will soon be applied.

There were two cases of amputation—one of the thigh, the other of the leg; both cases were admitted after the operation had been done. The latter case was instructive only in so far as it demonstrated the necessity for a second amputation, because of the immense and unwieldy flaps and useless stump; the necrosed ends of the bones had broken through the integument.

*Strumous Disease of the Knee—Amputation—Conical Stump.*

J. K., a boy ten years of age, of decidedly scrofulous diathesis, had been troubled with swelling of the knee during several years. The joint discharged through numerous long narrow tracts, above and below the patella, an ill-conditioned, watery, purulent fluid. The limb and the patient were much emaciated, and there were many scars of old fistulous tracts around the joint. Amputation, under ether, was done by the circular method, just above the condyles of the femur, Esmarch's bandage being used.

The stump is a conical one, the granulating end of the bone can be felt at its apex; the skin is retracted unevenly, and about one-fourth of the stump is a raw and granulating surface. This result was unexpected, and was due not to the operative procedures, but to the peculiar condition of the skin as impaired by the cicatricial tissue about the joint. Dr. Baxter remarks, that in a similar case he would allow more flap and amputate higher up on the limb, as the tissues show a tendency to retract and contract like the cicatrical tissue sequent upon burns.

*Esmarch's Bloodless Method.*

Dr. Baxter does not look upon this method with entire approval. In two cases of amputation of the lower extremity, in which this bandage was used, there was phlebitis, attended by the usual symptoms, and several abscesses in the limb. He is aware that such trouble might have occurred without the use of the bandage, but does not regard these attacks as coincidences, but as due to the method used. He has also had a patient suffer from numbness and pain in the limb, with partial disability, after using the bandage, which had been applied just below the elbow. Another objection to this method is, that pulmonary congestion is caused by expelling so much of the blood from a limb, the size of the leg and thigh, for example. There is a great deal of blood in this extremity, perhaps one-fifth of that in the entire circulation. Where does this blood go, when squeezed out by the rubber bandage? May it not be unsafe to use it in patients with weak pulmonary or cerebral vessels, and cause a congestion, which, in addition to that commonly attendant upon the use of anæsthetics, might be disastrous.

Finally, of this method of Esmarch, Dr. Baxter thinks that no rubber tubing is necessary, as the rubber bandage can so be adjusted as to answer every purpose.

*Excisions of Joints.*

The practice at this hospital is to limit this operation solely to the elbow and shoulder. A successful case of compound comminuted fracture was cited, where all of the right humerus had been removed, except its distal two inches. The man can now write, lift weights, and bring the forearm upon the chest, at right angles to the body.

---

***Rush Medical College.*** June 6, 1874. Clinic for Nervous Diseases, by Dr. WALTER HAY. *Multiple Cerebro-Spinal Sclerosis.*

The patient, a man of about thirty-five years of age, exhibits no aberration of sensibility, but there is a slight loss of motor power; hears on the left side only; has strabismus and impairment of sight. There are sharp, darting, hot pains along the spine, extending down the left leg; the muscles are soft and poorly nourished; action of bladder and bowels sluggish. The temperature of the left side of the body is lowered, due to irritation of the vaso-motor nerves.

In this patient there is a sclerosis—a hyperplasia of connective tissue of the spinal cord, and relative atrophy of the motor fibres; nutrition impaired, but no atrophy of nerve cells. The optic nerve, at its root, is also in this case diseased. It is a sclerosis of the disseminated form, for there are three forms, the diffuse, the cortical, and the disseminated. In sclerosis the lesion is one of motility; and in walking, the toe first touches the ground; while in progressive locomotor ataxy, the lesion is one of sensibility; in walking, the heel first touches the ground. The conductors of motor impulses pass from the brain, along the cord, on the same side as the muscles to which the nerve filaments are distributed. The pathological condition is identical in sclerosis and ataxy, but in one, the lesion is of the anterior column of the cord; in ataxy, the lesion is of the posterior column. The treatment in this case has been the inverse galvanic current to the spine, and faradization of the muscles of the left lower extremity.

---

### *St. Joseph's Hospital.*

The somewhat remote location of this admirable institution prevents its receiving that attention and patronage at the hands of the people and profession which its excellent management and arrangements and attractive surroundings merit. Under the care of Sister Walberga and five sisters, the hospital has no superior in regard to its large, airy, well lighted, ventilated and heated wards, and well furnished private rooms. Its wide corridors and piazzas, and the extensive exposure to sunlight, promote greatly the cheerfulness and speedy convalescence of patients.

A recent case of considerable interest, was that of a man who fell into a vat of boiling soap. Every part of the man, particularly the right side of the body, was scalded, so much so that considerable of the soft parts of the right side sloughed off. The man was reduced to the utmost emaciation. A diarrhœa proved well nigh fatal; it was checked, after repeated trials of the usual medicines, by drachm doses of tr. catechu, to which fifteen minims of laudanum were added. Lint soaked in a liniment of olive oil, to a pint of which a drachm of carbolic acid was added, was the only treatment externally.

